# A situational analysis of human resource and non-communicable diseases management for community health workers in Chengdu, China: a cross-sectional study

**DOI:** 10.1186/s12913-023-09880-z

**Published:** 2023-10-13

**Authors:** Jinhua Chen, Guo Yu, Wei Li, Chunyan Yang, Xiaoping Ye, Dan Wu, Yijun Wang, Wen Du, Zhu Xiao, Shuqin Zeng, Honglin Luo, Xiuhua Li, Yuelei Wu, Shuyi Liu

**Affiliations:** 1 Department of General Practice, Chengdu first people’s hospital, Chengdu, 610041 China; 2 Shiyang Community Health Service Center, Chengdu Hi-tech Zone, Chengdu, 610041 China; 3 Zhonghe Community Health Service Center, Chengdu Hi-tech Zone, Chengdu, 610041 China; 4 Guixi Community Health Service Center, Chengdu Hi-tech Zone, Chengdu, 610041 China

**Keywords:** Non-communicable diseases, Community health workers, Human resource, Training

## Abstract

**Background:**

Non-communicable diseases (NCDs) pose a major challenge to health economic cost and residents’ health status. Community health workers (CHWs) are the gatekeeper of primary health care.

**Objective:**

This study aimed to conduct a situational analysis of current human resource and requirements of NCDs-related training among CHWs in Chengdu with regard to address to understand the suggestions for improvement of challenges and barriers.

**Methods:**

A descriptive online cross-sectional survey was conducted among CHWs (doctors and nurses) from 23 districts and counties in Chengdu. Sociodemographic and NCDs-related variables were collected. Univariate analysis and multiple response analysis were used to describe the characteristics of these variables.

**Results:**

711 doctors and 637 nurses completely responded. There were significant differences among gender, age, educational levels, professional title, working year, type of institution, urban circle and registration in general practice between doctors and nurses (*P* < 0.001). 60.6% of doctors were female, compared to 98.0% for nurses. 58.2% of doctors held a bachelor’s degree compared with 45.4% of nurses, while 48.3% of nurses held a junior college degree compared with 25.7% of doctors. Higher levels of professional title and registration in general practice were found in doctors compared with nurses. The proportions of NCDs’ category, NCDs-related roles and tasks, NCDs-related training contents and forms that CHWs have attend and hoped to gain more were significantly different between doctors and nurses (*P* < 0.001). In general, the proportions in nurses were much lower than those of doctors (*P* < 0.05). The top five diseases managed by CHWs were hypertension, diabetes, cerebrovascular disease, chronic respiratory diseases and mental diseases. The five most reported roles performed among doctors included the distribution of health education (91.4%), following up (85.9%), establishing archives (71.3%), medicine adjustment (64.7%) and treatment implementation (52.0%). The top three diseases managed by nurses were same with doctors. The top four and five tasks were contact with patients or health services (39.6%) and referral (16.6%) in nurses. Most CHWs had received primary and common diseases-related trainings, but they had few opportunities to study in a tertiary hospital (40.4% in doctors and 20.9% in nurses, respectively), attend domestic academic conferences (26.9% in doctors vs. 9.7% in nurses), and take part in training courses (44.9% in nurses). CHWs hoped that the above-discussed training contents and forms could be provided more in the future. Besides basic skills related trainings, some specific skills related trainings should be strengthened.

**Conclusion:**

The qualifications in doctors were much better than those of nurses. The roles performed by CHWs in NCDs management are varied form common and frequent disease management to subsequent follow up and supervision. CHWs hope to receive more desired and oriented trainings. There is a need for building capacity of CHWs, optimizing and defining CHWs’ role, facilitating postgraduate medical education support and strengthening multidisciplinary collaboration would be effective in NCDs management.

Non-communicable diseases (NCDs), like cardiovascular diseases (CVD), cancers, chronic obstructive respiratory diseases and diabetes, are important restrictive factors for global public health. Due to the gradual transition of the spectrum of the disease, the ageing population, the increasing exposure to major NCDs risk factors and the rapid urbanization, the proportion of death caused by NCDs was increased from 68% (38 million) in 2012 [1] to 71% (40.5 million) in 2016 [2]. The burden is worse in low and middle-income countries (LMICs), where 78% of all NCD deaths occur and the death rate is about 1.6 times higher than in high-income countries [1]. The burden is worse in low and middle-income countries (LMICs), where 78% of all NCD deaths occur [3] and the death rate is about 1.6 times higher than in high-income countries [1]. Furthermore, the data from the World Economic Forum and Harvard University showed that NCDs are likely to cost the world economy $47 trillion over the next 20 years, revealing three-quarter of world gross domestic product (GDP) and surpassing the cost of the financial crisis worldwide [4]. Also, the prevalence of NCDs pose a major challenge to Chinese health system. According to the national data, NCDs in China were responsible for 86.6% of all deaths and 70% of the total health expenditure in 2015 [5]. The World Health Organization (WHO) statistics reported that 6.6 million deaths in China caused by NCDs in 2015, ranking top in the world [6]. In order to alleviate the threat from NCDs, the “National Basic Public Health Service Program” (NBPHSP) that includes both population based and group-specific health services (children, women, the elderly and NCDs patients) was implemented in 2009 when a major health-care reform was launched [7], and universal health coverage (UHC) agenda with reducing the financial burden of patients. Subsequently, Healthy China 2030 integrated health policies or strategies so as to improve overall health among Chinese population, and also laid stress on NCD prevention and control. Meantime, China has explored and developed four major models of chronic disease management (CDM) including family doctor contract service, community health service, internet plus healthcare model, and outpatient service management in public hospitals. The integration and utilization various medical resources not only contribute to prevent and manage chronic diseases but also provide people with a diverse, accessible and continuous health care.

Chronic disease management (CDM) is a comprehensive integration of primary care resources and policy guarantee, health systems, technology-enabled clinical data information management, and a well-organized health service managing collaborated procedure. It provides at different contents of NCDs and their risk factors, comprising early screening and intervention, regular and continuous testing, evaluation and intervention of medical behavior and process, following-up and health education, and referral process in community [8, 9]. Community health workers (CHWs) have become a mainstay of human resources for NCDs management in community. A CHW-led, interdisciplinary-enabled NCDs care model can feasibly screen high-risk factors and implement lifestyle interventions, lower disease complications, reduce hospitalization, cut down health expenditures and effectively improve health outcomes and quality of life [10–15]. Currently, family doctor contract services established by signing the contract of the service aimed to build a long-term and better medical cooperation between family doctors and the residents was carried out as a means of developing community health services. The implementation of family doctor contract services has improved patients’ perceived community primary care quality, and could help multi-agency collaboration. In China, despite efforts to address NCDs management in community, this issue is perpetuated by low capability and supply mismatch, low patient trust, heavy workload, inefficient referral system, inadequate numbers of trained and qualified human resources [16–20]. A necessary first step toward tackling the problem is to characterize the existing evidence base, and to determine opportunities and priorities for future improvement.

CHWs are divided into two categories according to community health service needs and the composition of family doctor team, and refer to professional CHWs (including doctors, nurses and health technicians) and non-medical professional workers (such as nutritionists, rehabilitation therapists, health service facilitators, and administrators) [21–24]. In China, CHWs have a broad composition including the employees who work in community health institutions and have been on the job for more than half a year [25]. Doctors and nurses are the main body of CHWs. Doctors and nurses are oriented towards their community’s needs and emphasize their practical availability to their community. The primary health care organization appears still largely based on a traditional doctor-centred model, while nurse is regarded as a team-mate to share the workload, to fulfill a range of responsibilities in health prevention, promotion, treatment, follow-up, and rehabilitation [26–28]. Having nurses take on tasks that are generally managed by doctors (nurse-doctor substitution and collaboration) may help to address unbalanced distribution of human resources and doctor shortages, increase people’s access to health care, achieve equal or better health outcomes, and improve the quality and continuity of care [28–30]. Challenges and barriers to the implementation of primary health care strategies are being solved step by step to provide substantial guarantees for ensuring that community health workers shoulder their gatekeeper responsibility to protect public and population health as well as deepen the reform of primary health-care services. Adequate and professional medical training of CHWs could improve their working ability and confidence, and satisfy their perception on NCDs management [31, 32]. On the demand side, if the health care providers’ needs and preferences are not taken into account, it is probably more circuitous to achieve to universal health and improve their work competency. Consequently, in this study we examined:(1) the compositional characteristics of community health workers in Chengdu by doctor and nurse, including the diversities in gender, age, working years, education level and professional title, (2) the relevant NCDs work they were engaged in, (3) the needs for NCDs training.

## Methods

### Sample and setting

To effectively evaluate CHWs’ status and needs, we selected Chengdu as the investigation site. Chengdu is the provincial capital city of Sichuan Province, and its permanent resident population was about 21.9 million in 2021. Furthermore, it has achieved a GDP of 1.99 trillion RMB in 2021. Given its geographic division and economic development, it is comprised of 23 districts and counties, and then it is divided into three urban circles, including Qingyang district, high tech zone, Dayi county and Pujiang county, and so forth. A descriptive cross-sectional study was conducted from 10 January to 17 January 2022, by means of online questionnaires conducted on an online survey platform called WenjuanXing administered to CHWs (doctor and nurse). Every 2 institutions from 23 districts and counties were selected. As a result, a total of 46 centres and 1,629 participants recruited in this study using multi-stage random cluster sampling. Considering the timeliness and validity of the investigation, the centre’s administration team member was responsible for the supervision, guidance and consultation of filling in the questionnaire. Eventually, excluding the missing records and extreme values, 1,348 participants completely responded anonymously (effective response rate: 82.75%). This study was approved by the ethics committee of Chengdu first people’s hospital. All subjects in this study were voluntary and expressed informed consent prior to the questionnaires.

### Instruments

#### Sociodemographic variables

Demographic and working characteristics collected included gender, age, educational level, professional title, type of institution, working year, urban circle and registration in general practice.

Gender was divided as ‘male’ and ‘female’. Age was categorized as ’20–30 years old’, ’31–40 years old’, ’41-50years old’, and ‘>50 years old’. Educational levels were divided into ‘secondary/ high school’, ‘junior college’, ‘bachelor’s, and ‘master’s, or above’. Professional title was classified into ‘primary title’, ‘middle title’, and ‘vice-senior title, or above’. Type of institution were divided as ‘township health center’, ‘second-class hospital or above’, ‘community health service center’ and others. Working year was divided as ‘≤5 years’, ‘6–9 years’, ‘10–15 years’, ‘16–20 years’ and ‘>21years’. Urban circle was categorized as ‘the first urban circle’, ‘the second urban circle’, and ‘the third urban circle’.

#### NCDs variables

The survey questionnaire was projected by the authors, which was guided by a combination of the government documents, previous research experience and the clinical investigator’s practice experience. In this section, there were two components in the questionnaire: (1) CHW’s current practices on NCDs management, and (2) facilitators and barriers to delivering NCD-related training. In brief, this part explored perception about: CHWs’ involvement to the responsibilities and implementation of NCD daily work, and attendance at obtainable in-service courses or training on NCDs management.

The questionnaire presented with multiple choice questions for an initial phase where CHWs were observed conducting their daily activities. Regarding their roles, CHWs were asked the following questions: 1) “What are NCDs that you manage?’ ,2) ‘What are your jobs in the prevention and treatment of NCDs?’ On the other hand, the participants’ experience of NCDs’ trainings, as well as their further expectations on trainings were observed by answering: What kind of NCDs training, training form, training content did you attend, and do you want to participate in, respectively?

#### Statistical analysis

All data analysis were conducted using IBM SPSS Statistics, version 25 for Windows (Microsoft, USA). The characteristics of variables were presented as percentages and arithmetic means and standard deviation. Analysis initially was performed with Chi-square test for frequency and reason of visit. Chi-square test was conducted to identify demographic and working characteristics between doctors and nurses. Multiple response analysis was performed to calculate the proportion of NCDs-related choices by occupation, and then the variables with a high ratio ranked top were selected, Subsequently, Chi-square test was performed to examine the difference of these variables between two groups. A two-tailed *P*-value < 0.05 was considered to be statistically significant.

## Results

### Socio-demographic characteristics of CHWs

The study population included 1348 participants, 711 doctors and 637 nurses. The participants’ characteristics were summarized in Table 1. 60.6% of doctors were female, compared to 98.0% for nurses (*P* < 0.001). 46.1% of doctors were aged at 31–40 years, by contrast 48.8% of nurses were aged at 20–30 years. Doctors had a higher level of qualification than their nurse counterparts (*P* < 0.001): 58.2% of doctors held a bachelor’s degree compared with 45.4% of nurses, while 48.3% of nurses held a junior college degree compared with 25.7% of doctors. Higher levels of professional title (*P* < 0.001) and registration in general practice (*P* < 0.001) were found in doctors compared with nurses. There were significant differences in working year, institutions and urban circle between two groups (*P* < 0.001).

**Table 1:** Socio-demographic characteristics of community health workers (n = 1348)

	Doctors(n = 711)	Nurses (n = 637)	*P*
**Gender**			0.000
Male	280(39.4%)	13(2.0%)	
Female	431(60.6%)	624(98.0%)	
**Age(years)**			0.000
20–30	114(16.0%)	311(48.8%)	
31–40	328(46.1%)	236(37.0%)	
41–50	206(29.0%)	78(12.2%)	
>50	63(8.9%)	12(1.9%)	
**Education**			0.000
secondary/ high school	79(11.1%)	40(6.3%)	
junior college	183(25.7%)	308(48.4%)	
bachelor’s	414(58.2%)	289(45.4%)	
master’s or above	35(4.9%)	0(0%)	
**Professional title**			0.000
primary title	336(47.3%)	444(69.7%)	
middle title	296(41.6%)	174(27.3%)	
vice-senior title or above	79(11.1%)	19(3.0%)	
**Type of institution**			0.000
township health center	256(36.0%)	198(31.1%)	
community health service center	370(52.0%)	396(62.2%)	
second-class hospital or above	51(7.2%)	42(6.6%)	
others	34(4.8%)	1(0.2%)	
**Working year**			0.000
≤ 5	76(10.7%)	130(20.4%)	
5–10	145(20.4%)	227(35.6%)	
10–15	179(25.2%)	149(23.4%)	
15–20	96(13.5%)	52(8.2%)	
≥ 20	215(30.2%)	79(12.4%)	
**Urban circle**			0.000
the first urban circle	280(39.4%)	341(53.5%)	
the second urban circle	200(28.1%)	193(30.3%)	
the third urban circle	231(32.5%)	103(16.2%)	
**Registration in general practice**			
yes	457(64.3%)	45(7.1%)	0.000
no	254(35.7%)	592(92.9%)	

### Roles and duties of CHWs related to NCDs

**Fig. 1:**
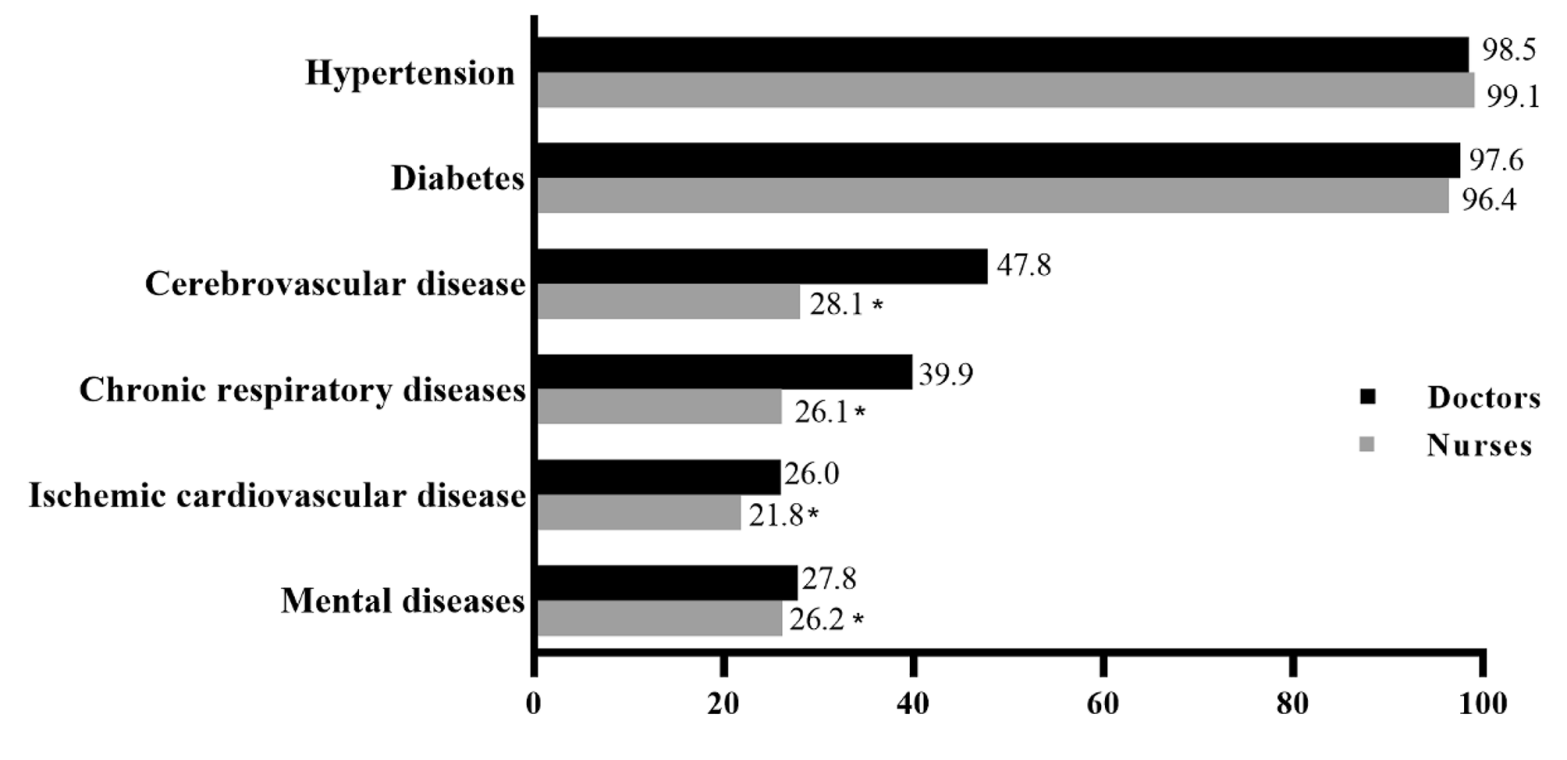
Distribution and difference of non-communicable diseases managed by doctors and nurses in the community, ^*^P<0.05

CHWs offered a diversity of responses on their roles in the management and care of NCDs (shown in Fig. 1). The order of the top five diseases managed by CHWs (both doctors and nurses) were hypertension, diabetes, cerebrovascular disease, chronic respiratory diseases and mental diseases. Significantly, there was a high proportion of mental diseases managed by CHWs (27.8% in doctors, 26.2% in nurses). Basically, doctors and nurses were involved with the same diseases management. But, the proportion of diseases managed by doctors and nurses was significant different (*P* < 0.001). Further analysis showed that the distribution of cerebrovascular disease, chronic respiratory diseases, ischemic cardiovascular disease, and mental diseases between doctors and nurses was remarkable different (*P* < 0.05).

**Fig. 2:**
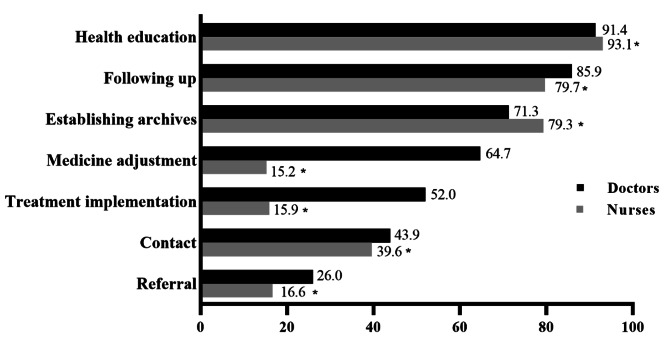
Distribution and difference of non-communicable diseases-related roles or duties performed by doctors and nurses, *P<0.05

The NCDs-related duties were presented in Fig. 2. The five most reported roles performed among doctors included the distribution of health education (91.4%), following up (85.9%), establishing archives (71.3%), medicine adjustment (64.7%) and treatment implementation (52.0%). Nevertheless, there were some differences in nurses group. The sequence order among nurses was health education (93.1%), following up (79.7%), establishing archives (79.3%), contacting with patients or health services (39.6%) and referral (16.6%), of those last two duties also were the routine duties for doctors (43.9% and 26.0%). There was significant different in the proportion of NCDs-related roles performed by doctors and nurses (*P* < 0.001). Further analysis showed that the percentage of all above mentioned jobs undertaken by doctors and nurses was remarkable different (*P* < 0.05). Except for health education and establishing archives, doctors were committed to more routine duties than nurses.

### Trainings related to NCDs

**Fig. 3:**
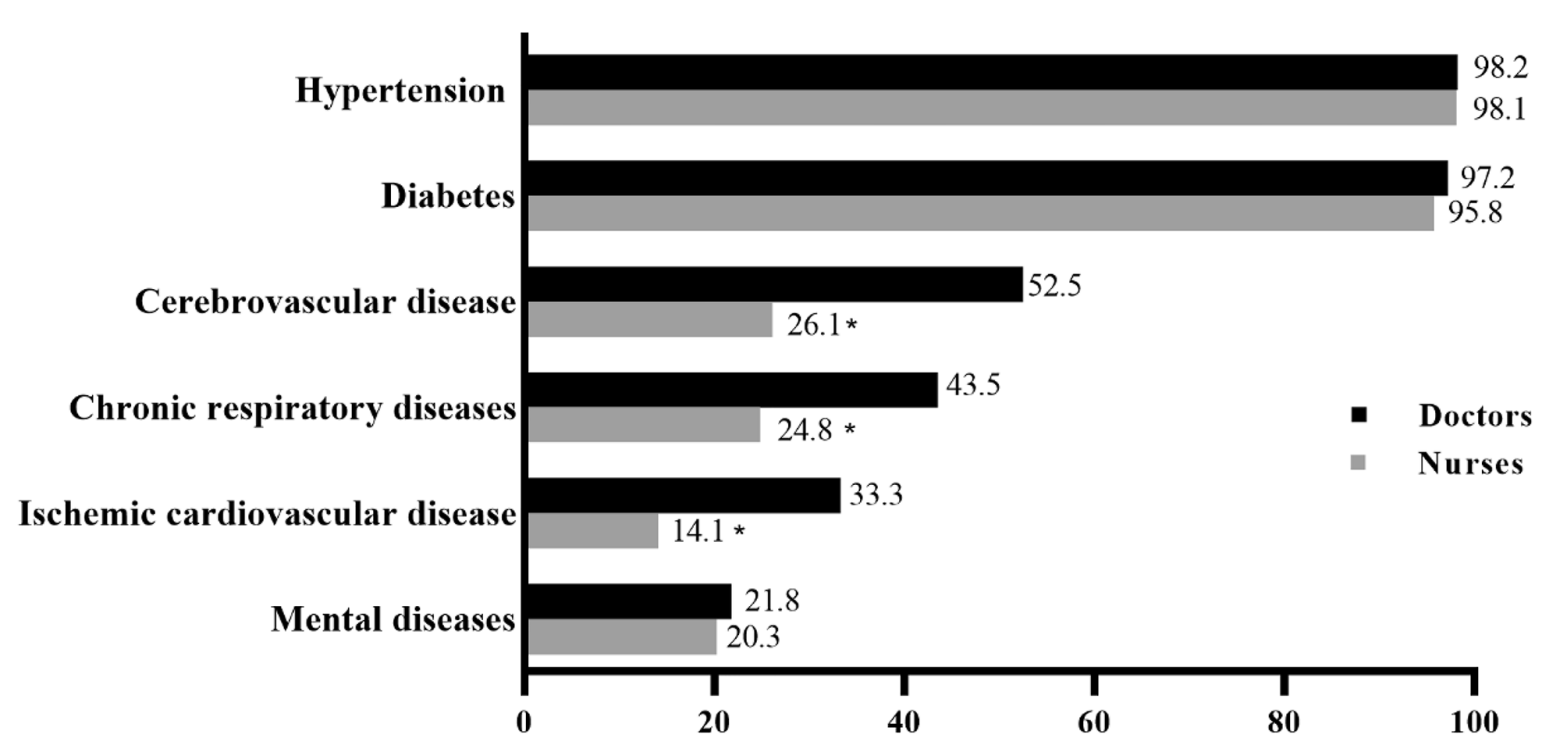
Frequency and difference of NCDs-related trainings between doctors and nurses, ^*^P<0.05

As showed in Fig. 3, the frequency of NCDs-related training that doctors have attended was highest on hypertension (98.2%), followed by diabetes (97.2%), cerebrovascular disease (52.5%), chronic respiratory diseases (43.5%), ischemic cardiovascular disease (33.3%) and mental diseases (21.8%). Generally, nurses also mainly had been involved with the same trainings, but they had low opportunities compared with doctors. Of 637 nurses interviewed, only 166 (26.1%) reported having received cerebrovascular disease-related training, and of those, 158 (24.8%) participated in chronic respiratory diseases-related training. There was a significantly different in the proportion of NCDs-related trainings attended by doctors and nurses (*P* < 0.001). Further analysis showed that doctors had a higher percentage of training on cerebrovascular disease, chronic respiratory disease, and ischemic cardiovascular disease than nurses (*P* < 0.05).

**Fig. 4:**
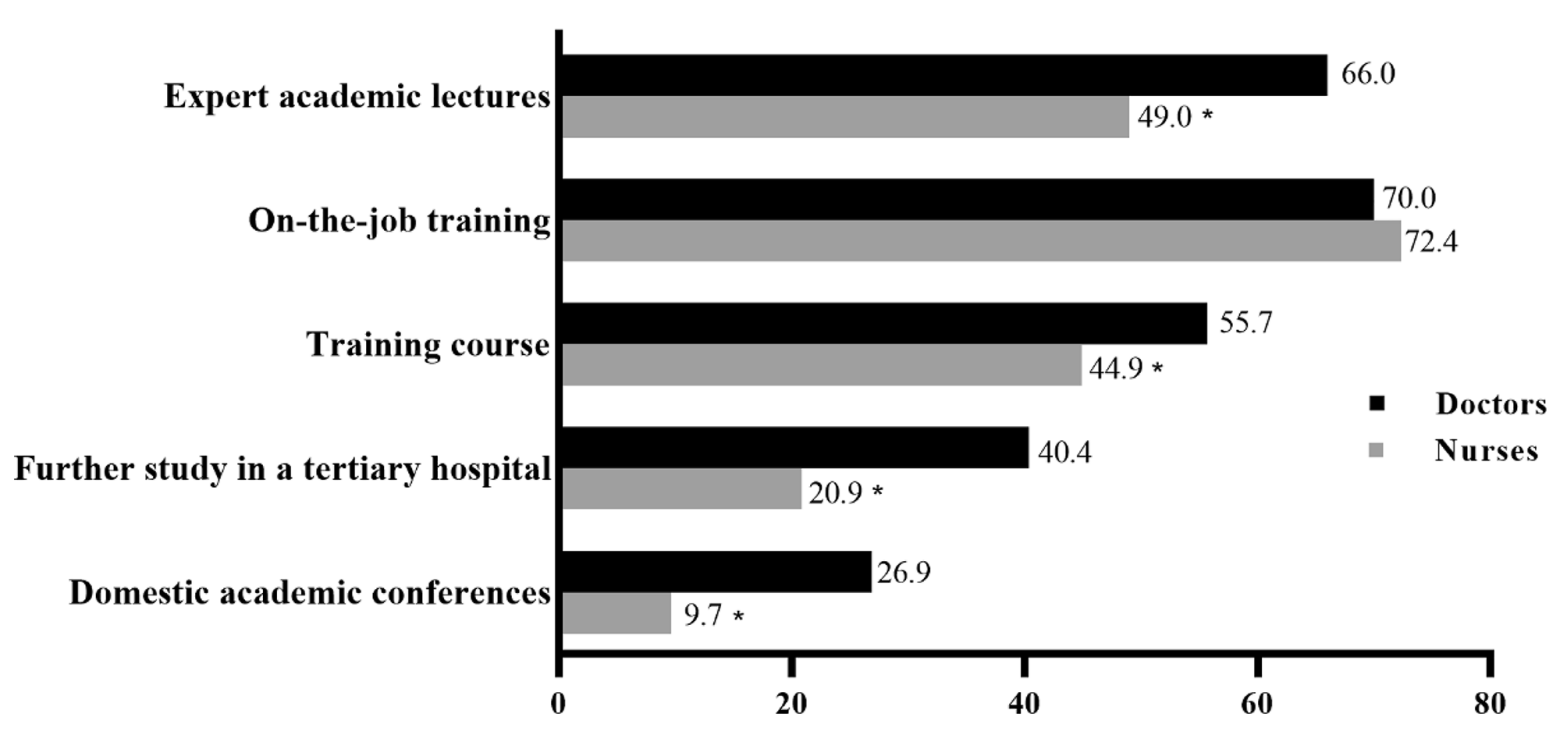
Distribution and difference of non-communicable diseases-related training forms between doctors and nurses, ^*^P<0.05

The five forms with the highest frequency of NCDs- related trainings that doctors have been attended were as follows: on-the-job training (70.0%), expert academic lectures (66.0%), training course (55.7%), further study in a tertiary hospital (40.4%), and domestic academic conferences (26.9%). Although nurses had the same experience with their doctor colleagues, in comparison the proportion of training form among nurses was considerably low. More specifically, less than half of nurses (n = 286, 44.9%) took part in training courses, a comparative low ratio of nurse participants (n = 133,20.9%) had studied in a tertiary hospital. Analysis displayed that the distribution of training form received by doctors and nurses was significant different (*P* < 0.001), and doctors had a higher percentage of training on expert academic lectures, training course, further study in a tertiary hospital, and domestic academic conferences than nurses (*P* < 0.05). These descriptions are shown in Fig. 4.

**Fig. 5:**
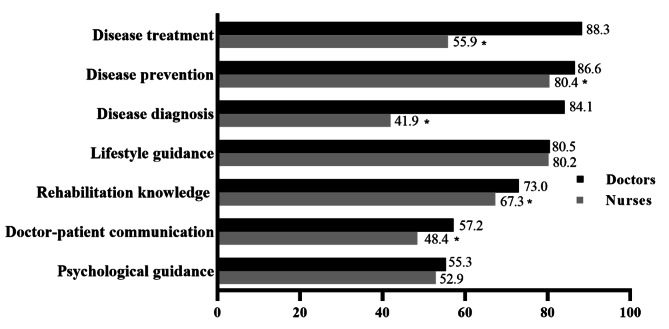
Distribution and difference of non-communicable diseases-related training contents that were expected to be added in the future between doctors and nurses, ^*^P<0.05

The desirable NCDs-related training contents were displayed in Fig. 5. It was hoped that disease treatment (88.3%), disease prevention (86.6%), disease diagnosis (84.1%), lifestyle guidance (80.5%), rehabilitation knowledge (73.0%), doctor-patient communication (57.2%), and psychological guidance (55.3%) were added into the NCDs-related training contents among doctors. Similarly, nurses also hoped these training contents could be added into their career planning. Disease prevention (80.4%), lifestyle guidance (80.2%), and rehabilitation knowledge (67.3%) were top three in nurses’ training list. Analysis displayed that the distribution of future training contents between doctors and nurses was significant different (*P* < 0.001), and doctors had a higher percentage of disease treatment, disease prevention, disease diagnosis, rehabilitation knowledge, and doctor-patient communication than nurses (*P* < 0.05).

**Fig. 6:**
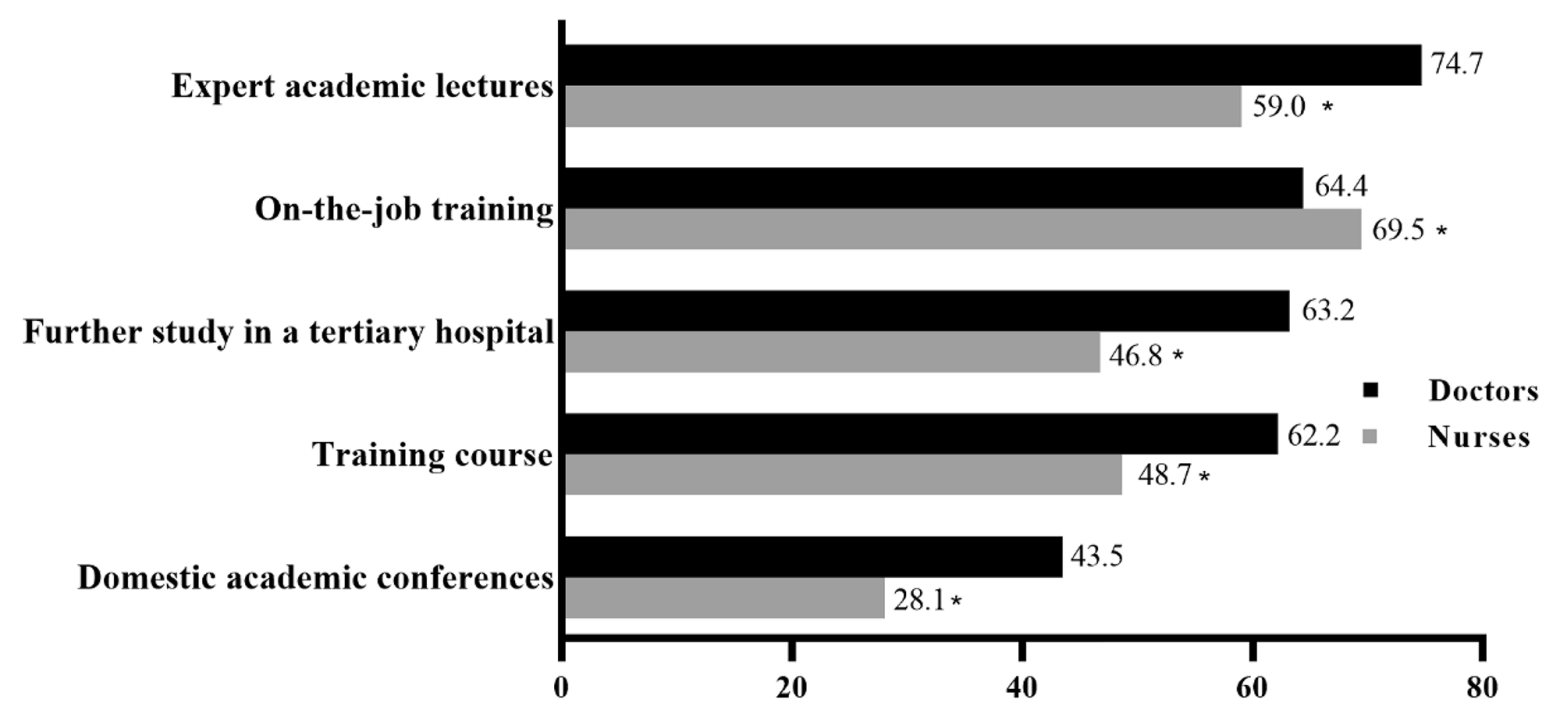
Distribution and difference of non-communicable diseases-related training forms that were expected to be added in the future between doctors and nurses, ^*^P<0.05

The vision of NCDs- related training form (presented in Fig. 6) in doctors was that expert academic lectures (74.7%), on-the-job training (64.4%), further study in a tertiary hospital (63.2%), training course (62.2%), and domestic academic conferences (43.5%) were orientated into their future training forms. Meanwhile, nurses also hoped that the forms mentioned above could be provided in their future career. However, nurses had a lower percentage of expert academic lectures, training course, further study in a tertiary hospital, and domestic academic conferences than doctors (*P* < 0.05).

## Discussion

CHWs-based NCDs management play a key role in developing a continuous, effective and integrated care delivery system. CHWs who have frequent contact with patients are well positioned to provide counselling, as well as reliable sources of information from patients’ perspective. This large survey of China’s CHWs investigates the current primary care workforce and shows it is predominantly staffed by young female employees, with about 60% of doctors holding a bachelor degree or higher or registered as a GP. The proportion of higher education level and registration in GP was relatively low in nurses. In many primary health care programmes, females are the preferred gender because of the type of tasks required [33]. Considering the inequality of salary and continuous medical education, workers with a higher education level prefer to a general hospital rather than community health services. A study based on the individual-level data showed that a comparative low proportion of registered CHWs worked in primary health care [20]. In primary health-care system, many CHWs were previously either public health physicians or specialist who converted to being GPs and were allowed to keep up to three specialties on their registration. Several previous studies showed that the fairness on distribution of primary health resources was poorer in China, due to geographical and economic disparity [26, 34, 35], which in turn leads to increasing inequalities between the rich and the poor with regard to health and the economic burden of health care. A shortage of CHWs could be a prominent barrier to scaling-up quality improvement of NCDs management and improve the satisfaction of patients on chronic care. It is necessary to issue the policies highlighting the human resources and encouraging higher continuing education, which is contributed to resolve the aforementioned challenges. In the meantime, nurse-doctor substitution has been perceived as an effective and available strategy to alleviate workforce shortages, to improve the quality and continuity of health care, and to reduce human capital investment [29, 36–38]. The measures could be generalized to our districts, but it should be taken the context-specific and tailored circumstances into consideration, such as type of service, reciprocal respect and collaboration between physicians and nurses, proper resources, and good referral systems [30]. Furthermore, encouraging the experts from tertiary hospitals to participate in CHWs’ daily work has been promulgated as the national policy to narrow the substantial gap in the overall primary health system and to increase CHWs’ competency.

We found that the top five NCDs managed by CHWs were hypertension, diabetes, cerebrovascular disease, chronic respiratory diseases and mental diseases. The disease composition of NCDs reflected that CHWs are more often dealing with primary and common diseases, which were substantially different from those worked in the tertiary hospitals. Significantly, mental diseases which are often ignored are considerable common at community. A number of published studies reported that anxiety and depressive disorders were common among patients with chronic diseases. Hypertension, diabetes, cerebrovascular disease, and chronic kidney disease were found to be associated with depressive symptoms [39–41]. Furthermore, a large-scale epidemiological survey conducted in China was revealed the relationship of NCDs with psychological symptoms: multimorbidities and a course of disease within 1 year or more than 5 years were associated with a higher risk of stress, anxiety and depression [42]. Besides, somatization was a frequent co-morbid psychiatric symptom among NCDs patients [43]. The prevalence of mental health problem is expected to pose a threat to the healthcare system in China. Accordingly, a modest amount of effort is still required to address screening, diagnosis and management of these diseases.

Doctors and nurses who are regarded as the main workforce in the community provide a broad range of services ranged from the distribution of health education to following up, establishing archives, medicine adjustment, treatment implementation, contacting with patients or health services and referral. These roles are a clear indication of CHWs’ efforts in assisting patients to achieve a better health outcome. However, shouldering various of responsibilities can reduce their ability to be efficient and job satisfaction [44, 45]. Notably, CHWs are at the forefront of preventing and controlling the virus during COVID-19 pandemic, which causes them to assume increasing responsibility and multitasking works, resulting in stressed and depressed [46]. Thus, it is necessary to define the clear role of CHWs, which not only meet their current needs but also contribute to community development. In addition, this study reported that the role performed by doctors and nurses was slightly different. Although, the primary health care model is based on traditional physician-centric care, mutual trust and cooperation among them on primary care might drive NCDs management to a promising prospect.

In our study, it was demonstrated that training-related NCDs that CHWs have received was matched with the NCDs managed by them. Studies have showed that training could boost CHWs’ working ability and self-confidence to be qualified for NCDs management [47–51]. In this survey, it revealed that CHWs were not satisfied with the contents and forms of training in our region. From the perspective of doctors, it was most hoped that disease treatment, disease prevention, and disease diagnosis were added into the NCDs-related training contents. It was slightly different among nurses that disease prevention, lifestyle guidance, and rehabilitation knowledge were top three requirements. Additionally, psychological guidance and doctor-patient communication were also expected to be included in the training list. CHWs were more likely to attend more on-the-job training, expert academic lectures, training course, further study in a tertiary hospital, and domestic academic conferences. The majority of CHWs have not obtained enough postgraduate or continuing medical educations and trainings to meet the basic work requirements and demands [52, 53]. As a result of this barrier, CHWs have limited ability to prevent, diagnose and treat NCDs. Improving risky lifestyle behaviours (smoking, alcohol abuse, sedentary life and unhealthy diet) is the most important strategy in preventing, reducing morbidity and burden of NCDs. However, counselling on lifestyle guidance from CHWs is insufficient and partly due to a lack of training. As doctor-patient communication is a subtle but important part of medical process. A bad doctor patient relationship is responsible for misunderstandings and conflicts and decreasing health care quality. Despite the advantages of effective and efficient doctor-patient communication on health care, patient-centred care and communication should be enhanced among CHWs. Furthermore, due to lack of professional mental health skills, CHWs were incompetent to screen and diagnose mental illness, and to provide psychological counseling [54]. In addition, eliminating discrimination against mental illness from CHWs was an intervention to improve the quality of mental health services [55, 56]. Hence, relevant trainings should be developed to enrich CHWs’ professional knowledge about mental illness. Notably, the training program should be based on CHWs’ expectations so as to make CHWs mastered the key concepts, as well as included follow-up support and feedback that could strengthen more transfer of knowledge after training.

Some limitations should be considered in this survey. First, the sample population was relatively small, limiting the generalisability of the findings to other cultures and geographic regions in China. Second, this study used a cross-sectional design and no causal relationships can be speculated. Third, the study was restricted to the preferences of doctors and nurses, which may limit specificities to other professions and insights from the service recipients’ perspective. Fourth, participants made choices based on these selected variables, other variables had not been fully considered in the study. Furthermore, quantitative study is needed to improve these limitations.

In this study, we found that doctors and nurses are disproportionately at community in Chengdu: doctors had a higher level of qualification, professional title and registration in general practice compared with nurses. Although doctors and nurses are exposed to the same primary care management environment, doctors rather than nurses are perceived as the principal implementer in community-orientated primary care. The trainings on NCDs among CHWs are insufficient and not tailored. Considering the context and characteristics of CHWs, specific and standardised NCD training should be provided. Significantly, except for the basic and imperative job requirements, trainings-related mental illness and doctor-patient communication should be enhanced. To strengthen the roles of CHWs and to promote NCDs management programmes, more work-based learning opportunities such as on-the-job training and further study in a tertiary hospital should be provided and matched with the preferences and expectations of CHWs.

## Data Availability

The data and materials in this study are available from the corresponding author on reasonable request.
